# Supplementary material for: A soft selective sweep during rapid evolution of gentle behaviour in an Africanized honeybee
Source: Nat Commun. 2017 Nov 16;8:1550. doi: 10.1038/s41467-017-01800-0 (PMC5688081; doi:10.1038/s41467-017-01800-0)
Supplement: Supplementary file 1 — Supplementary Information [file 41467_2017_1800_MOESM1_ESM.pdf]

SUPPLEMENTARY TABLES

Supplementary Table 1. Gene Ontology terms found to be significantly overrepresented in a hypergeometric test of 250 genes with extreme Rsb values.

GO Term	Description	Enrichment score	# of genes	% of gene list	p-value
GO:0016021	integral component of membrane	4.85	80	32	3.84E-05
GO:0005509	calcium ion binding	1.78	13	5.2	0.0052
GO:0008188	neuropeptide receptor activity	1.62	6	2.4	0.0018
GO:0007186	G-protein coupled receptor signaling pathway	1.62	9	3.6	0.0082
GO:0007623	circadian rhythm	1.51	6	2.4	0.0087
GO:0045211	postsynaptic membrane	1.38	7	2.8	6.42E-04

SUPPLEMENTARY FIGURES

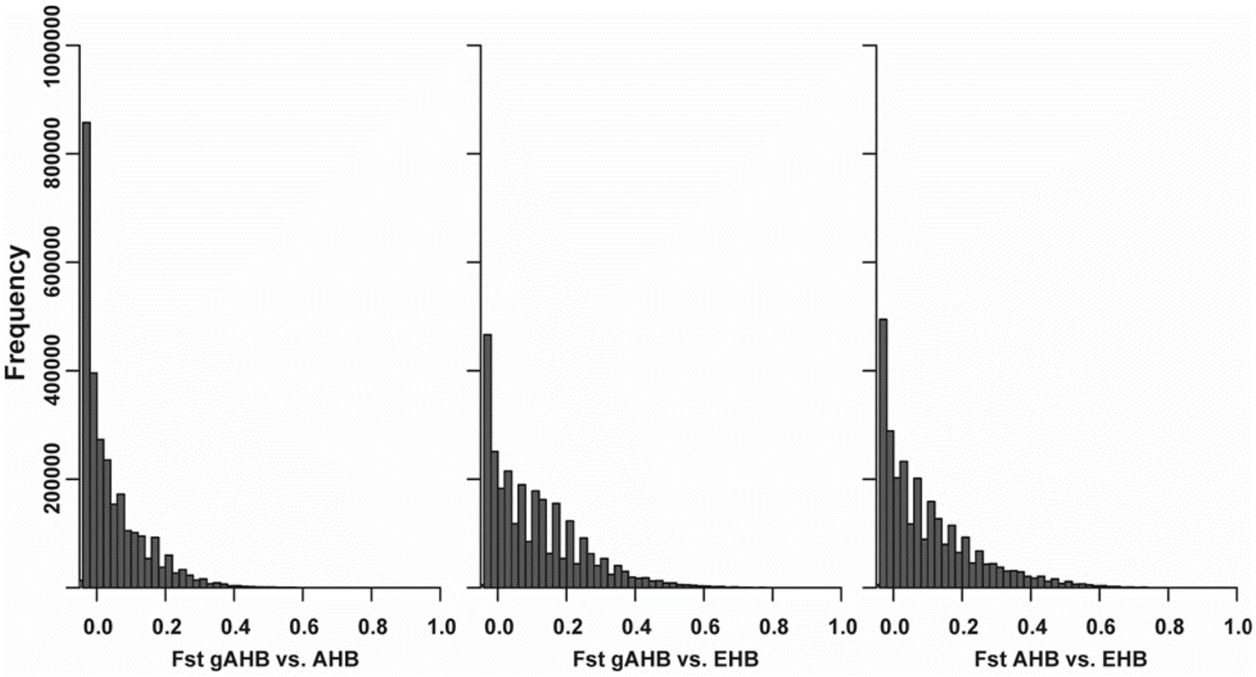

Supplementary Figure 1. Distribution of F_{ST} signals in each of the three pairwise population comparisons. Diagrammatic summary of F_{ST} distribution in each of the pairwise comparisons between gAHB, AHB, and EHB. Clear pattern is evident where gAHB and AHB share similar frequencies (low fixation values) and agrees with gAHB as derived from AHB.

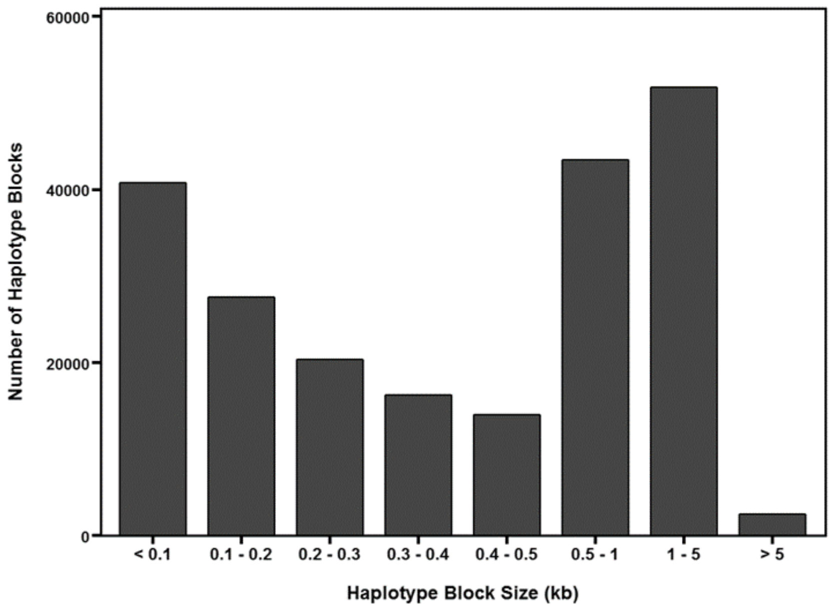

Supplementary Figure 2. Haplotype Block Size Distribution. Binned distribution of haplotype block sizes. The majority of the haplotype blocks spanned $\leq$ 5,000 base pairs, concordant with known recombination rates across the honeybee genome[1].

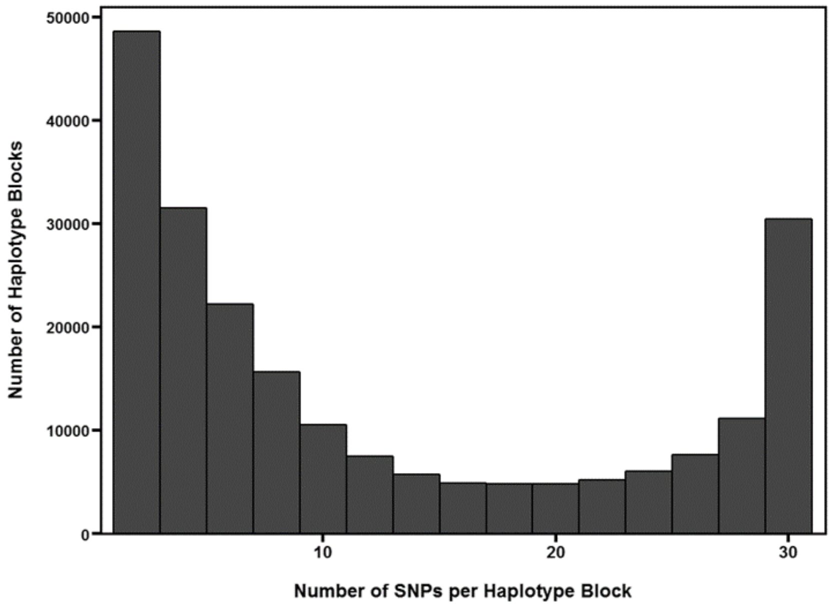

Supplementary Figure 3. SNPs within Haplotype Blocks. The graph shows the bi-modal distribution of SNPs across haplotype blocks defined by GERBIL[2]. Overall most blocks contained either fewer than 15 SNPs or greater than 28 SNPs.

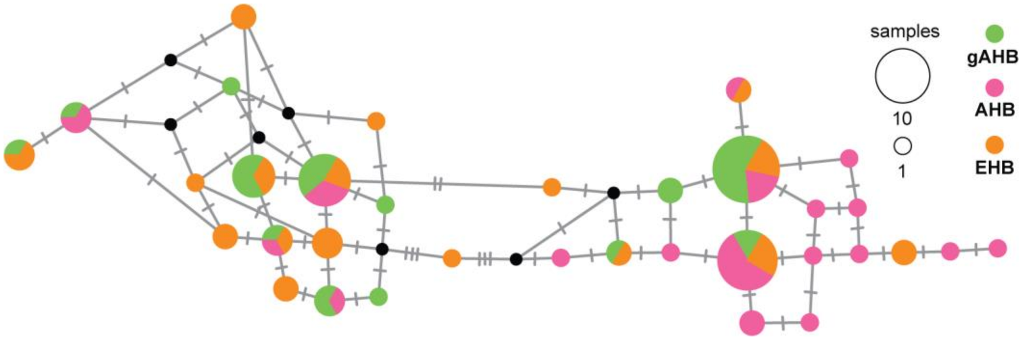

Supplementary Figure 4. Median-Joining Network of the *csd* Gene. The diagram visualizes an allelic network constructed from non-synonymous SNPs within the coding sequence of the *csd* gene. Colors correspond to population membership, while sizes of the circles are proportional to the sample size. Thatch marks denote mutational steps in the sequence separating the alleles. Black circles denote median vertex of the network.

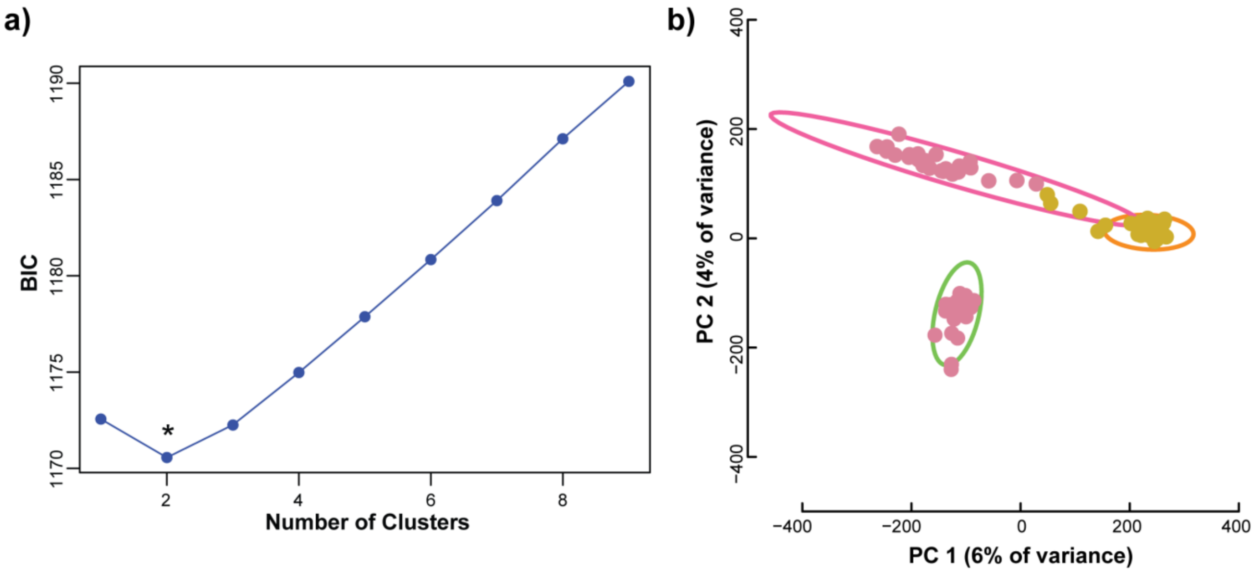

Supplementary Figure 5. Analysis of broad genetic groups across gAHB, AHB, and EHB. (a) Optimal number of clusters was identified through successive K-means clustering analysis, an optimal k = 2 (star) was determined by this process. **(b)** PCA of just the gAHB, AHB, and EHB samples from this study colored by cluster membership gold for EHB-like, pink for AHB-like. Ellipses represent 95% confidence interval of the populations, and color traces correspond to population membership as in Fig.3b (green for gAHB, orange for EHB, and pink for AHB).

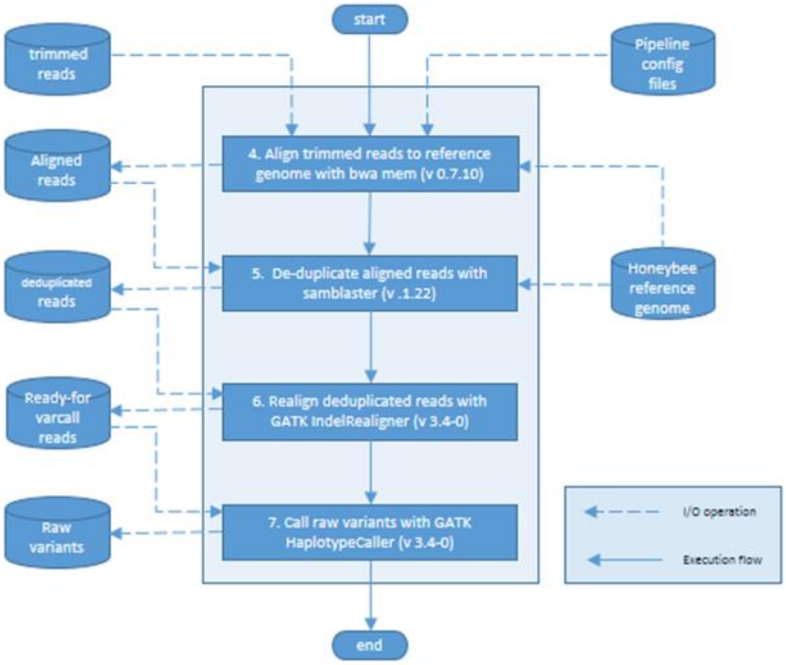

Supplementary Figure 6. Diagrammatic workflow illustrating our variant calling pipeline. The chart provides workflow and corresponding computational tools utilized in our variant calling pipeline. This process resulted in individual sample SNP profiles, which were later processed in a downstream joint variant calling step using the GATK-GenotypeGVCFs tool.

Supplementary Note 1: Sample bash language code detailing key steps in the variant calling pipeline developed and implemented in this study

Step 1. For each sample, de-duplicated, sorted `**.bam` file was realigned using a two-step process. First, the file was processed with GATK (version 3.4-0)[3] -RealignerTargetCreator which generates a list of intervals. Second, this list was utilized by GATK (version 3.4-0)[3] -IndelRealigner along with the de-duplicated, sorted `**.bam` files to generate the realigned `**.bam` files used for later variant calling steps

```
java -Xmx8g -Xms1024m -Djava.io.tmpdir=$RealignDir -jar $gatk_dir/GenomeAnalysisTK.ja
r \
  -R $ref_local \
  -I $AlignDir/${SampleName}.wdups.sorted.bam \
  -T RealignerTargetCreator \
  -nt $thr \
  -o ${SampleName}.realignTargetCreator.list

java -Xmx8g -Xms1024m -Djava.io.tmpdir=$RealignDir -jar $gatk_dir/GenomeAnalysisTK.ja
r \
  -R $ref_local \
  -I $AlignDir/${SampleName}.wdups.sorted.bam \
  -T IndelRealigner \
  -targetIntervals ${SampleName}.realignTargetCreator.list \
  -o ${SampleName}.realigned.bam
```

Step 2. Once all the sample realigned `**.bam` files were produced, each was provided to GATK (version 3.4-0)[3] -HaplotypeCaller with the parameters listed below for individual variant calling.

```
java -Xmx6g -Xms1g -Djava.io.tmpdir=./ -jar $gatk_dir/GenomeAnalysisTK.jar \
 -T HaplotypeCaller \
 -R $ref_local \
 -I $RealignDir/${SampleName}.realigned.bam \
 --emitRefConfidence GVCF --variant_index_type LINEAR --variant_index_parameter 12800
0 \
 -gt_mode DISCOVERY \
 -A Coverage -A FisherStrand -A StrandOddsRatio -A HaplotypeScore -A MappingQualityRa
nkSumTest -A QualByDepth -A RMSMappingQuality -A ReadPosRankSumTest \
 -stand_call_conf 30 \
 -stand_emit_conf 30 \
 --sample_ploidy 1 \
 -nt 1 -nct $thr \
 -o ${SampleName}.rawVariants.vcf
```

Step 3. Resulting `**.gvcf` files along with the reference genome were used as input for GATK (version 3.4-0)[3] -GenotypeGVCFs tool which conducted joint variant calling across all the samples.

```
java -Xmx6g -Xms1g -jar /home/apps/gatk/gatk-3.5/GenomeAnalysisTK.jar \
 -T GenotypeGVCFs \
 -R ./genome.fa \
 -V ./gvcfidx.list \
 --sample_ploidy 1 \
 -stand_call_conf 30 \
 -stand_emit_conf 30 \
 -o ./2016_Jan_19_ProductionRun1_allsamples_mvcall.vcf
```

Supplementary Note 2: sample bash language code detailing variant filtering steps conducted following variant calling.

Step 1. For unplaced scaffold exclusion vcftools version 0.1.14[4] was implemented. Input was the unfiltered **.vcf derived from the joint calling step which contained only biallelic SNPs, and an index listing all the unplaced scaffolds.

```
vcftools --vcf ./2016_Jan_29_ProductionRun_1_SNPs_only.recode.vcf --out ./2016_Jan_29
_ProductionRun_1_SNPs_only_unplfilter --exclude-positions ./2016_Jan_29_ProductionRun
_1_snpinfo_unplfilter.idx --recode --recode-INFO-all
```

Step 2. NUMT region exclusion was also conducted using vcftools version 0.1.14[4]. Target NUMT regions were previously identified using NUCmer version 3.0[5,6] (see Methods). The index of regions identified by our approach was provided as input along with the **.vcf file filtered in Section 2.1 to exclude NUMTs.

```
vcftools --vcf ./2016_Jan_29_ProductionRun_1_SNPs_only_unplfilter.recode.vcf --out ./
2016_Jan_29_ProductionRun_1_SNPs_only_draft1 --exclude-bed  ./NUMT.bed --recode --rec
ode-INFO-all
```

Step 3. A two-step bash script was used to apply quality filters to the `**.vcf` file. GATK (version 3.4-0)[3] –VariantFiltration was initially implemented to apply a filter for those SNPs not

meeting any of the criteria detailed in the code below (also see Methods). Once the filter was in place GATK (version 3.4-0)[3] –SelectVariants tool was used to extract only those SNPs that passed all filters.

```
java -Xmx16g  -Djava.io.tmpdir=$tmpdir -jar $gatk_dir/GenomeAnalysisTK.jar \
-T VariantFiltration \
-R ./Amel_4.5_scaffolds.fa \
-V ./ 2016_Jan_29_ProductionRun_1_SNPs_only_draft1.recode.vcf \
--filterExpression "AN < 72.0" --filterName "AN_FAIL" \
--filterExpression "QUAL < 1376 || QUAL > 75300" --filterName "QUAL_FAIL" \
--filterExpression "DP < 288 || DP > 24330" --filterName "DP_FAIL" \
--filterExpression "QD < 2.0 || MQ < 4.0 || MQRankSum < -12.4 || ReadPosRankSum < -8.
0 || FS > 60.0 || SOR > 3.0" --filterName "SNP_HARD_FAIL" \
-o ./2016_Jan_29_ProductionRun_1_SNPs_only_draft1.qcfiltered.vcf

if [ ! -f ./2016_Jan_29_ProductionRun_1_SNPs_only_draft1.qcfiltered.vcf ];then
echo "filtered file does not exist, aborting."
exit
fi

if [ -f ./2016_Jan_29_ProductionRun_1_SNPs_only_draft1.qcfiltered.vcf ];then

java -Xmx16g  -Djava.io.tmpdir=$tmpdir -jar $gatk_dir/GenomeAnalysisTK.jar \
-T SelectVariants \
-R ./Amel_4.5_scaffolds.fa \
-V ./2016_Jan_29_ProductionRun_1_SNPs_only_draft1.qcfiltered.vcf \
-o ./2016_Jan_29_ProductionRun_1_SNPs_only_draft1.qcfiltered.excluded.vcf \
-ef

fi
```

Supplementary Note 3: sample R code describing analysis of genetic structure in target populations.

Initial analysis of genetic structure of the sampled populations (gAHB, AHB, EHB) was conducted using the `adegenet`[7] package in R[8]. A principal component analysis (PCA) was applied to the whole data set. The PCA of the data along with the data itself was used as the input for the `find.clusters` function, which conducts a sequential k-means clustering algorithm to determine the optimal number of genetic clusters (k) present in the data.

```
#read in the data
snpgl <- readRDS("./dapc/snp_genlight") #note that the genlight object is specific to
adegenet and was constructed prior to analysis form the SNP 0,1 matrix.

snpgl #preview

##  /// GENLIGHT OBJECT /////////
##
##  // 90 genotypes,  2,808,570 binary SNPs, size: 295.8 Mb
##  5212483 (2.06 %) missing data
##
##  // Basic content
##    @gen: list of 90 SNPbin
##    @ploidy: ploidy of each individual  (range: 1-1)
##
##  // Optional content
##    @ind.names:  90 individual labels
##    @loc.names:  2808570 locus labels
##    @loc.all:  2808570 alleles
##    @chromosome: factor storing chromosomes of the SNPs
##    @position: integer storing positions of the SNPs
##    @pop: population of each individual (group size range: 30-30)
##    @other: a list containing: elements without names

#conduct the PCA
snpgl.pca <- glPca(snpgl, nf = 90, n.cores = 20) #nf retains the specified number of
PCs while n.cores allows parallel processing

#find the optimal number of clusters (k) in our data set
snpgl.fclust <- find.clusters(snpgl, n.pca = 90, stat = "BIC", max.n.clust = 4, choos
e.n.clust = F, criterion = "smoothNgoesup", glPca = snpgl.pca, n.iter = 10000) #we co
nstrained our search from k=1 to k=4, and performed 10,000 iterations.
```

Supplementary Note 4: sample R code detailing analysis of population genetics statistics (Fst, Rsb).

Calculation of population statistics F_{ST} and *Rsb* were conducted in R[8], using custom code provided here and in our repository (`https://github.com/HPCBio/Honeybee-VariantCalling`).

Step 1. F_{ST} calculation required data re-formatting into a binary matrix (2,808,570 SNPs by 90 samples; see below). We also embedded metadata in the sample names for later use.

```
snp.mat <- snpgr$snpcall #extracting the SNP 0,1 matrix from the Genomic Ranges objec
t housing the data set
dim(snp.mat) #dimensions of the full matrix

## [1] 2808570      90

head(colnames(snp.mat)) #column names identify samples and source populations

## [1] "110MEX_WHAIPI005600-66_AHB" "115MEX_WHAIPI005599-64_AHB"
## [3] "11PR_WHAIPI005633-80_gAHB"  "12PR_WHAIPI005634-81_gAHB"
## [5] "1PR_WHAIPI005630-74_gAHB"   "20PR_WHAIPI005635-85_gAHB"
```

Step 2. Each pairwise calculation of F_{ST} was conducted using two of the three populations in the sample matrix highlighted in Section 4.1, for each pair the source data matrix was sub-sectioned, then the function `WC_Haplotype_Fst_Function.R` was applied.

```
snp.mat <- snp.mat[, -(grep("_AHB", colnames(snp.mat)))] #this excludes the AHB popul
ation, thus the comparison is gAHB v. EHB

dim(snp.mat) #dimensions of the new matrix

## [1] 2808570      60

pop <- gsub("(\\S+)_(\\S+)_(\\S+)","\\3", colnames(snp.mat)) #extract the observed po
pulation memberships to use as input in Fst calculation formula

head(pop) #preview

## [1] "gAHB" "gAHB" "gAHB" "gAHB" "gAHB" "gAHB"

source("./WC_Haplotype_Fst_Function.R") #call the Fst function

obs <- wc_hapfst(geno = snp.mat, subpop = pop) #create a vector to house the observed
Fst values
head(obs) #preview

## [1] 0.36563262  0.24316748  0.17001909  0.12955869  0.04424779 -0.03961595
```

Step 3. Validation of F_{ST} was achieved through permutations our strategy first constructed a matrix of pre-randomized sample population membership, then used these iteratively along with the sub-sectioned matrix.

```
pop_boot <- fread(input = "./gAHBvEHB_pop", sep = "\t", header = F, verbose = F, show
Progress = F, data.table = F) #our matrix of randomized population memberships

head(pop_boot) #preview
##      V1   V2   V3   V4   V5   V6   V7   V8   V9  V10  V11 V12  V13  V14 …
## 1 gAHB  EHB  EHB  EHB  EHB  EHB gAHB gAHB  EHB  EHB  EHB EHB gAHB  EHB
## 2  EHB gAHB gAHB gAHB gAHB  EHB gAHB  EHB gAHB  EHB gAHB EHB gAHB  EHB
## 3  EHB  EHB  EHB  EHB gAHB gAHB gAHB  EHB gAHB gAHB  EHB EHB gAHB gAHB
```

```
## 4 gAHB gAHB  EHB gAHB  EHB gAHB  EHB  EHB gAHB  EHB  EHB EHB gAHB  EHB
## 5 gAHB gAHB gAHB  EHB  EHB gAHB  EHB  EHB  EHB  EHB  EHB EHB gAHB gAHB
## 6 gAHB  EHB  EHB gAHB  EHB gAHB gAHB  EHB  EHB  EHB  EHB EHB gAHB gAHB
##  …

#permutation steps
count <- rep(0, nrow(snp.mat)) #first we generated an empty counter with an entry for
each SNP
R <- seq(1000) #then set the number of permutations


#lastly we iterate
for (i in R) {
  tmp = wc_hapfst(geno = snp.mat, subpop = pop_boot[i,]) #calculate Fst with the rand
omized population membership
  tmp = as.numeric(obs <= tmp) #compare whether the permuted Fst is greater than or e
qual to our observed Fst (1=TRUE)
  tmp[is.na(tmp)] <- 0 #this intermediary step removes NA values which are provided b
y the function when the populations are monomorphic
  count <- count + tmp #add the instances in this iteration to the counter
}

head(count) #preview the counts of instances

## [1]   1   2  24  14 132 792
```

Step 4. The counter generated in the approach detailed in Section 4.3 was used to derive the p-value for our F_{ST} calculations as recommended by Weir[9] in his derivation of haplotype F_{ST} calculations.

```
#p-value derivation
p_val <- count / max(R) #proportion of permuted Fst values higher than or equal to th
e observed Fst p-value
fdr<-p.adjust(p_val, method = "fdr") #family-wise error rate correction


head(fdr) #preview

## [1] 0.00454545 0.00645161 0.048979592 0.03333333 0.203076923 0.82500000
```

Step 5. Calculation of *Rsb* required re-formatting our data set.

```
snp.map <- fread(input = "./hap_files/snp.map", sep = "\t", header = T, verbose = F,
showProgress = F, data.table = F) #mapping file


head(snp.map) #preview

##              nm      chr pos ref alt
## 1 Group1.1_552 Group1.1 552   T   A
## 2 Group1.1_576 Group1.1 576   C   T
## 3 Group1.1_585 Group1.1 585   A   G
## 4 Group1.1_610 Group1.1 610   G   A
```

```
## 5 Group1.1_612 Group1.1 612   G   A
## 6 Group1.1_631 Group1.1 631   T   G

exfl <- fread(input = "./hap_files/Group1.1.gahb.hap", sep = "\t", header = F, verbos
e = F, showProgress = F, data.table = F) #example of a haplotype file format


head(exfl) #preview

##   V1 V2 V3 V4 V5 V6 V7 V8 V9 V10 V11 V12 V13 V14 V15 V16 V17 V18 V19 V20 …
## 1  T  A  T  T  T  T  T  A  T   T   T   T   T   A   T   T   A   T   T   A
## 2  C  T  C  C  C  C  C  T  C   C   C   C   C   T   C   C   T   C   C   T
## 3  G  G  A  A  A  A  G  G  A   G   G   G   A   G   A   A   G   A   A   G
## 4  G  G  A  G  G  A  G  G  A   G   G   G   G   G   G   G   G   A   G   G
## 5  G  G  G  G  G  A  G  G  A   G   G   G   G   G   G   G   G   G   G   G
## 6  T  T  T  T  T  G  T  T  G   T   T   T   T   T   T   T   T   T   T   T
## …
```

Step 6. For each of the 909 files generated in our re-formatting step, we applied the `scan_hh` function from the rehh package which extends the haplotype from each SNP in both directions until a threshold haplotype decay (0.1) is reached then calculates the area under the curve.

```
scaf <- unique(snp.map$chr) #extract the unique scaffolds

head(scaf) #preview

## [1] "Group1.1" "Group1.2" "Group1.3" "Group1.4" "Group1.5" "Group1.6"

#for each unique scaffold
for (i in scaf) {
  hap_file = paste(i, ".gahb.hap", sep = "") #identify the corresponding gAHB file
  data <- data2haplohh(hap_file = paste("./hap_files/", hap_file, sep=""), "./snp.map
", haplotype.in.columns = TRUE, recode.allele = TRUE, chr.name = i) #converts data to
the haplohh format
  res <- scan_hh(data, threads = 7) #calculate scaffold- and population- specific met
rics

  #the following checks whether the first item in the sequence is also the first scaf
fold in the genome (Group1.1)
  if (i == "Group1.1") {wg.res.gahb <- res} #if it is, it creates a copy of the res v
ector
  else {wg.res.gahb <- rbind(wg.res.gahb, res)} #if it is not, it appends the data in
the res vector to the already existing object

  #the process was also iterated over the AHB samples
  hap_file = paste(i, ".ahb.hap", sep="")
  data <- data2haplohh(hap_file = paste("./hap_files/", hap_file, sep = ""), "./hap_f
iles/snp.map", haplotype.in.columns = TRUE, recode.allele = TRUE, chr.name = i)
  res <- scan_hh(data, threads = 7)
  if (i == "Group1.1") {wg.res.ahb <- res} else {wg.res.ahb <- rbind(wg.res.ahb, res)
}

  #and and the EHB samples
```

```r
  hap_file = paste(i, ".ehb.hap", sep = "")
  data <- data2haplohh(hap_file = paste("./hap_files/", hap_file, sep = ""), "./hap_files/snp.map", haplotype.in.columns = TRUE, recode.allele = TRUE, chr.name = i)
  res <- scan_hh(data, threads = 7)
  if (i == "Group1.1") {wg.res.ehb <- res}else{wg.res.ehb <- rbind(wg.res.ehb, res)}
  print(i)
}

head(wg.res.gahb) #example preview of final, concatenated output

##                    CHR POSITION    freq_A     iHH_A    iHH_D
## Group1.1_552 Group1.1      552 0.7333333  251.8957 8749.819
## Group1.1_576 Group1.1      576 0.7333333  251.8957 8749.819
## Group1.1_585 Group1.1      585 0.5000000  628.4289 2093.183
## Group1.1_610 Group1.1      610 0.7333333 1123.8122 1051.330
## Group1.1_612 Group1.1      612 0.8333333  796.4600 3556.750
## Group1.1_631 Group1.1      631 0.8333333  796.4600 3556.750
##              iES_Tang_et_al_2007 iES_Sabeti_et_al_2007
## Group1.1_552            846.1608              355.8410
## Group1.1_576            846.1608              357.6617
## Group1.1_585           1220.0863              371.0330
## Group1.1_610           1108.7981              489.8420
## Group1.1_612            879.0017              526.5168
## Group1.1_631            879.0017              527.8099
```

Step 7. The concatenated output from the `scan_hh` function was utilized in the `ies2rsb` function, which calculated final statistics.

```r
wg.rsb.gahbvahb <- ies2rsb(wg.res.gahb, wg.res.ahb, "gAHB", "AHB") #calculate Rsb

head(wg.rsb.gahbvahb) #preview

##                    CHR POSITION Rsb (gAHB vs. AHB)
## Group1.1_552 Group1.1      552         -0.5482708
## Group1.1_576 Group1.1      576         -0.5482708
## Group1.1_585 Group1.1      585         -0.9781813
## Group1.1_610 Group1.1      610         -1.6007694
## Group1.1_612 Group1.1      612         -1.8005389
## Group1.1_631 Group1.1      631         -1.8426425
##              -log10(p-value) [bilateral]
## Group1.1_552                   0.2339547
## Group1.1_576                   0.2339547
## Group1.1_585                   0.4841465
## Group1.1_610                   0.9608715
## Group1.1_612                   1.1440232
## Group1.1_631                   1.1845469

summary(wg.rsb.gahbvahb[, 3]) #summary of ln(Rsb)

##    Min. 1st Qu.  Median    Mean 3rd Qu.    Max.    NA's 
## -4.5330 -0.6380  0.0000  0.0974  0.7335  5.3710       3
```

```
summary(exp(wg.rsb.gahbvahb[, 3])) #summary of Rsb

##      Min.   1st Qu.    Median      Mean   3rd Qu.      Max.      NA's
##   0.01075   0.52830   1.00000   1.96800   2.08200 215.20000         3
```

SUPPLEMENTARY REFERENCES

1. Beye, M. *et al.* Exceptionally high levels of recombination across the honey bee genome. *Genome Res.* **16,** 1339–44 (2006).
2. Kimmel, G. & Shamir, R. GERBIL: Genotype resolution and block identification using likelihood. *Proc. Natl. Acad. Sci.* **102,** 158–162 (2005).
3. DePristo, M. A. *et al.* A framework for variation discovery and genotyping using next-generation DNA sequencing data. *Nat. Genet.* **43,** 491–8 (2011).
4. Danecek, P. *et al.* The variant call format and VCFtools. *Bioinformatics* **27,** 2156–2158 (2011).
5. Delcher, A. L., Phillippy, A., Carlton, J. & Salzberg, S. L. Fast algorithms for large-scale genome alignment and comparison. *Nucleic Acids Res.* **30,** 2478–2483 (2002).
6. Kurtz, S. *et al.* Versatile and open software for comparing large genomes. *Genome Biol.* **5,** R12 (2004).
7. Jombart, T. adegenet: a R package for the multivariate analysis of genetic markers. *Bioinformatics* **24,** 1403–1405 (2008).
8. R Core Team. R: A language and environment for statistical computing. (2016).
9. Weir, B. *Genetic Data Analysis II: Methods for Discrete Population Genetic Data*. (Sinauer Associates Inc., 1996).
