# Supplementary material for: A soft selective sweep during rapid evolution of gentle behaviour in an Africanized honeybee
Source: Nat Commun. 2017 Nov 16;8:1550. doi: 10.1038/s41467-017-01800-0 (PMC5688081; doi:10.1038/s41467-017-01800-0)
Supplement: Supplementary file 3 — Description of Additional Supplementary Files [file 41467_2017_1800_MOESM3_ESM.pdf]

File Name: **Supplementary Data 1**
Description: **Annotation summary of genes containing at least one candidate haplotype block.** The data set includes relevant information labels for each of the 35 genes found to contain at least one haplotype block identified in our analysis. Specific information listed includes the gene name, the name of its closest *Drosophila* homologue (where available), the number of candidate haplotypes, the number of candidate SNPs within the haplotypes, ontology and pathway categories associated with each gene (where available), and a listing of prior aggression studies where the gene has been identified as associated with the behavior. A more detailed description for each specific annotation label is provided in the first sheet of the document.

File Name: **Supplementary Data 2**
Description: **Summary report of alignment for each of the individual samples.** The data set provides a summary report of the alignment quality for each of the samples used in this study. Relevant metrics such as quality of alignment and percent mapped reads are provided alongside more specific alignment metrics.
